# Hypothesis: Possible influence of antivector immunity and SARS‐CoV‐2 variants on efficacy of ChAdOx1 nCoV‐19 vaccine

**DOI:** 10.1111/bph.15620

**Published:** 2021-07-31

**Authors:** Loris Zamai, Marco B. L. Rocchi

**Affiliations:** ^1^ Department of Biomolecular Sciences University of Urbino Carlo Bo Urbino Italy; ^2^ Gran Sasso National Laboratory (LNGS) National Institute for Nuclear Physics (INFN) L'Aquila Italy

**Keywords:** environmental conditions, immunity, influenza virus, physical prevention, SARS‐CoV‐2 variants, vaccine, viral vectors

## Abstract

The present work provides arguments for the involvement of anti‐vector immunity and of SARS‐CoV‐2 variants on the efficacy of ChAdOx1 nCoV‐19 vaccine. First, it is suggested that anti‐vector immunity takes place as homologous vaccination with ChAdOx1 nCoV‐19 vaccine is applied and interferes with vaccine efficacy when the interval between prime and booster doses is less than 3 months. Second, longitudinal studies suggest that ChAdOx1 nCoV‐19 vaccine provides suboptimal efficacy against SARS‐CoV‐2 Alpha variant, which appears to have an increased transmissibility among vaccinated people. At the moment, ChAdOx1 nCoV‐19 vaccine is able to reduce the severity of symptoms and transmissibility. However, if the vaccinated individuals do not maintain physical preventive measures, they could turn into potential spreaders, thus suggesting that mass vaccination will not quickly solve the pandemic. Possible consequences of SARS‐CoV‐2 evolution and of repeated anti‐SARS‐CoV‐2 vaccinations are discussed and adoption of an influenza‐like vaccination strategy is suggested.

**Abbreviations**
AU: arbitrary units
CI: confidence interval
GMR: geometric mean ratio
Ig: immunoglobulin
IQR: interquartile range
LD: low dose
NTD: N‐terminal domain
RBD: receptor binding domain
SD: standard dose
S‐D: single standard dose

## 1 ChAdOx1 nCoV‐19 VACCINE CLINICAL TRIALS

A vaccine is a special drug that people do not take every day but only once or a few times. It primes the immune system to fight off an infection. However, like any drug, vaccines can vary in probability of both effectiveness and side effects, and they can induce drug/vaccine resistance; benefits and risks that can differ depending on age, comorbidities and other genetic and/or environmental factors. The widespread mortality and morbidity associated with the COVID‐19 pandemic has induced the development of several vaccines (Kyriakidis et al., 2021), some of which have recently received emergency use authorisation. Among them, ChAdOx1 nCoV‐19 vaccine was authorised with a regimen of two standard doses (SDs) given with an interval of 4–12 weeks on the basis of the interim analysis data (Voysey et al., 2021b). Following regulatory approval, the optimal dose interval was assessed in a recent report through post hoc exploratory analyses (Voysey et al., 2021a). The ChAdOx1 nCoV‐19 vaccine consists of a replication‐deficient chimpanzee adenoviral vector containing the full‐length SARS‐CoV‐2 spike glycoprotein gene, which was tested across different studies (Folegatti et al., 2020; Ramasamy et al., 2021; Voysey et al., 2021b). Based on previous experience with ChAdOx1 MERS (van Doremalen, Haddock, et al., 2020), the vaccination studies (COV001‐UK, COV002‐UK, COV003‐Brazil and COV005‐South Africa) were initially designed to assess a single dose (5 × 10^10^ viral particles) of ChAdOx1 nCoV‐19 (Folegatti et al., 2020; Ramasamy et al., 2021; Voysey et al., 2021b), although other vaccination protocols consist of first (i.e., prime) and second (i.e., boost) doses. Differently from human virus‐vectored vaccines, for which pre‐existing anti‐vector immunity could reduce the vaccine immunogenicity, a chimpanzee adenovirus‐vectored vaccine can bypass this possibility. However, after the first (priming) dose, tere is the possibility to develop an anti‐vector immunity, which could inhibit the potency of the booster dose. Preliminary data showed that vaccination of rhesus macaques with a single dose of ChAdOx1 nCoV‐19 was able to protect against SARS‐CoV‐2 infection, indicating the efficacy of the single‐dose strategy (van Doremalen, Lambe, et al., 2020). However, once the studies were underway, the analysis of immune responses and other factors led to amendments to the trials including groups receiving different vaccination protocols in the analysis. Initially, low dose (LD) of viral particles (2.2 × 10^10^ viral particles) due to an inaccurate quantification of viral particles by spectrophotometric methods was administered, and further doses were adjusted to the SD (5 × 10^10^ viral particles), using a more accurate qPCR assay (Voysey et al., 2021b). Induction of both spike‐specific neutralising antibody titres and T cell responses has been shown to provide protection against viral infections in animal models (van Doremalen, Haddock, et al., 2020; van Doremalen, Lambe, et al., 2020), and the immunogenicity data from Phase 1 (COV001‐UK, begun on 23 April 2020) showed a substantial increase in SARS‐CoV‐2 spike neutralising antibodies (but not in interferon‐γ ELISpot T cell response to SARS‐CoV‐2 spike peptides) with a second dose of vaccine given after 28 days (Folegatti et al., 2020). Based on this observation, the trial protocols were modified to a regime of two doses administered 28 days apart (Folegatti et al., 2020). While it was ongoing, the above protocol changes were applied to a second study (COV002‐UK), which included participants who received a low dose (LD) of the vaccine (2.2 × 10^10^ viral particles) as their first dose and were boosted with a SD (3.5–6.5 × 10^10^ virus particles), called LD/SD group, and subsequently participants who were vaccinated with two SD vaccines (SD/SD group). The LD/SD cohort was enrolled between 31 May and 10 June 2020, whereas the SD/SD cohort (aged 18–55 years) was enrolled later from 9 June to 20 July 2020 (Voysey et al., 2021b). Boosting began on 3 August 2020, resulting in a longer gap between prime and booster vaccines in LD/SD cohort (median 84 days, interquartile range, IQR, 77–91) than for those in SD/SD cohorts (median 69 days, IQR 50–86) (Voysey et al., 2021b). Indeed, most participants in the LD/SD group received a second dose around 12 weeks after the first, whereas the interval between doses for the SD/SD group (target 28 days) was both lower and more heterogeneous because of an insufficient production of the vaccine (Voysey et al., 2021b). Differently, a trial in Brazil (COV003), which began on 23 June 2020, included a SD/SD group with the majority of participants receiving a second dose within 6 weeks of the first (median 36 days) (Voysey et al., 2021b). Finally, some participants who received a low first dose (originally planned as single‐dose cohort) chose not to receive the second dose and constituted a cohort of low single‐dose recipients (Voysey et al., 2021a; Voysey et al., 2021b). These situations provide the opportunity to analyse the vaccine efficacy of a single dose and the effect of different dose intervals. Unfortunately, there was no overlap in enrolment of participants in these cohorts, and participants of LD/SD cohort and single LD cohort were vaccinated (prime dose) before those of SD/SD cohort (Voysey et al., 2021b).

## 2 POSSIBLE INFLUENCE OF ANTI‐VECTOR IMMUNITY ON EFFICACY OF ChAdOx1 nCoV‐19 VACCINE

Interestingly, vaccine efficacy against symptomatic or asymptomatic disease in participants (COV002‐UK) who received a LD as their first dose of vaccine (LD/SD) was significantly higher than that of participants who received SD/SD vaccines (Voysey et al., 2021a,  2021b). Indeed, vaccine efficacies in LD/SD group was 90.0% (95% CI 67.4–97.0) and 58.9% (95% CI 1.0–82.9) against symptomatic and asymptomatic (evaluated by mean of weekly self‐swab) disease, respectively, whereas they were, respectively, 60.3% (95% CI 28.0–78.2) and 3.8% (95% CI −72.4 to 46.3) in SD/SD group (data cut‐off on 4 November 2020) (Voysey et al., 2021b), indicating that the two trial protocols produced significantly different protection from SARS‐CoV‐2 symptomatic and asymptomatic disease and transmission. Moreover, the SD/SD cohort in Brazil displayed a relatively low protection, 64.2% (95% CI 30.7–81.5), which was similar to vaccine efficacy of SD/SD UK cohort (60.3%). These surprising data might suggest that a low first dose would induce a longer and/or a higher SARS‐CoV‐2 immune protection. However, other factors such as dose interval are likely to be involved in determining the significant differences between LD/SD and SD/SD cohorts. In this regard, both the UK (COV002) and Brazil (COV003) SD/SD cohorts, which displayed relatively low vaccine efficacies against primary symptomatic COVID‐19, had shorter dose intervals than LD/SD cohort (Voysey et al., 2021b), suggesting that the longer dose intervals of LD/SD group might give higher protection. Notably, a subsequent analysis (data cut‐off on 7 December 2020) (Voysey et al., 2021a) revealed that when SD/SD group was restricted to those who received their vaccines more than 84 days (12 weeks) between the two doses (a dose interval similar to LD/SD group), vaccine efficacy of SD/SD cohort (81.3% [95% CI 60.3–91.2]) was similar to that of LD/SD cohort (80.7%, [95% CI 62.1–90.2]) (Voysey et al., 2021a). Moreover, ChAdOx1 nCoV‐19 vaccine had a higher efficacy in SD/SD group with a longer prime‐boost interval (vaccine efficacy 81.3% [95% CI 60.3–91.2] at ≥84 days) than in those with a short interval (vaccine efficacy 55.1% [95% CI 33.0–69.9] at <42 days), further suggesting that long (≥84 days) dose intervals give higher protection. However, 84 days of dose interval might increase the probability of infection between the two doses. In this regard, although anti‐SARS‐CoV‐2 spike IgG responses after a single SD of ChAdOx1 nCoV‐19 vaccine showed a decrease from the peak at Day 28 (median 5496 AU·ml^−1^ [IQR 2548–12,061] for participants aged 56–69 years and 9807 AU·ml^−1^ [IQR 5847–17,220] for participants aged 18–55 years) of 34% by Day 90 (geometric mean ratio [GMR] 0.66 [95% CI 0.59–0.74]), a single SD was efficacious (76.0% [95% CI 59.3–85.9]) against primary symptomatic (but not against asymptomatic) SARS‐CoV‐2 infection in the first 90 days after vaccination, with no significant waning of protection during this period, thus supporting the approach to delay second doses (Voysey et al., 2021a). As indicated in the report, participants were removed from the analysis of single‐dose efficacy at the time of their booster dose (Voysey et al., 2021a). However, most participants in the single‐dose analysis received a second dose within 90 days after the first dose. That means that the data analysed for participants from 22 to 90 days since the first dose were collected before the data cut‐off date indicated in the report (7 December 2020), possibly between August and October. Instead, the group of participants reaching 91 and 120 days since the first dose is likely to represent subjects who never received a second dose, for which vaccine efficacy was assessed at the data cut‐off date (7 December 2020). During this last 30‐day period, the vaccine efficacy of the single dose appeared to wane, reaching only 31.6% protection (95% CI −141.8 to 80.7). This is possibly due to a progressive decrease of anti‐SARS‐CoV‐2 spike IgG responses (64% by Day 180, GMR 0.36 [0.27–0.47]) from the peak at Day 28 and/or other factors (e.g., SARS‐CoV‐2 variants emerging during the month of November, see later). Altogether, the data suggested that a 3‐month dose interval provided better protection after a second dose without compromising protection in the period before the booster dose is administered. This conclusion was supported by immunogenicity data that showed that in both LD/SD and SD/SD cohorts, participants who received a second SD of vaccine more than 84 days after the first had anti‐SARS‐CoV‐2 spike IgG titres more than twofold higher than those who received the second dose within 42 days of their initial vaccination. Assuming there is a relationship between the humoral immune response and vaccine efficacy, this evidence suggested that long (≥84 days) dose intervals were more efficacious than shorter dose intervals and could induce a long protection from SARS‐CoV‐2 (Voysey et al., 2021a). These data were recently discussed in a report (Voysey et al., 2021a). However, the possible mechanism(s) underlying this observation was not discussed.

In this context, the likelihood of developing an anti‐vector immunity on homologous boosting has been raised and this eventuality could explain the reduced efficacy of the booster dose when it was administered earlier than 84 days after the first dose. Indeed, it is likely that immunity against the antigenic proteins of simian adenovirus vector tends to wane during the time (as well as that against spike proteins), providing a rational explanation of the increased anti‐SARS‐CoV‐2 spike IgG responses and the increased vaccine efficacy produced by delayed boosting.

## 3 POSSIBLE INFLUENCE OF UK SARS‐CoV‐2 VARIANT(S) ON EFFICACY OF ChAdOx1 nCoV‐19 VACCINE

**FIGURE 1:**
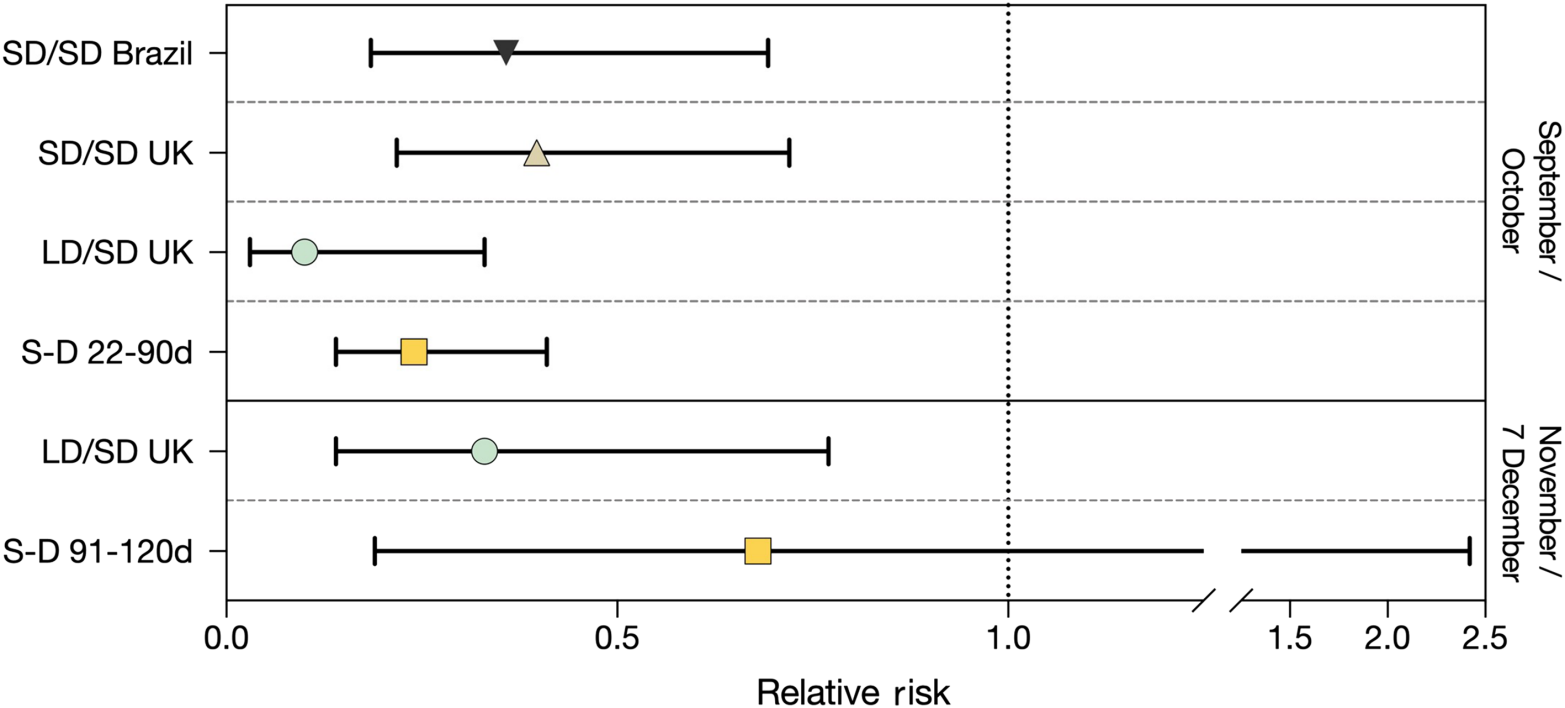
Relative risk of symptomatic infection and 95% CI in different groups of vaccine participants with different dose interval analysed in two different time periods (September/October vs. November/7 December 2020). SD/SD = two standard‐dose vaccine groups with shorter dose interval assessed in Brazil (median 36 days of dose interval) or United Kingdom (69 days of dose interval) during September/October (4 November cut‐off date). LD/SD = low‐dose prime plus standard‐dose boost groups with longer (median 84 days) dose interval assessed before (September/October) and after (November/7 December) 4 November. S‐D = single standard‐dose vaccine assessed between 22 and 90 days (22‐90d, corresponding to the period between September and October) or between 91 and 120 days (91‐120d, including the period between November and 7 December) after injection

Of note, comparison of vaccine efficacy data between the two different cut‐off dates for the participants in the LD/SD cohort (the only group that remained constant in numbers and therefore comparable in longitudinal analyses), the values may suggest a slight decrease of protection (Voysey et al., 2021a). Indeed, the relative risk of infection in LD/SD group at the first data cut‐off date (4 November 2020) was 0.10 (95% CI 0.03–0.33) and 0.41 (95% CI 0.17–0.99) for symptomatic and asymptomatic disease, respectively (Voysey et al., 2021b), whereas they were subsequently estimated to be, respectively, 0.19 (95% CI 0.10–0.38) and 0.51 (95% CI 0.28–0.93) at the second data cut‐off (7 December 2020) (Voysey et al., 2021a), possibly suggesting a slight decrease of vaccine efficacy during the last period of about a month. In this regard, at the first data cut‐off date (4 November 2020), within the LD/SD group, the symptomatic infected individuals were three in 1367 participants (0.2%) in the vaccinated group and 30 in 1374 participants (2.2%) in the control group. Instead, at the second data cut‐off date (7 December 2020), within the same LD/SD group, the symptomatic infected individuals were 10 in 1396 participants (0.7%) in the vaccinated group and 51 in 1402 participants (3.6%) in the control group. That means that during the time between the two data cut‐off dates (basically the month of November), there were seven symptomatic infections in 1393 participants (1396 minus three already infected) (0.50%) in the vaccinated group and 21 in 1372 participants (1402 minus 30 already infected) (1.53%) in the control group, which correspond to 0.33 (95% CI 0.14–0.77) of relative risk of symptomatic infection that is about three times higher than that of the first time period. Notably, the LD/SD cohort was enrolled between 31 May and 10 June 2020 (Voysey et al., 2021b), and most of them had a booster dose about 3 months later (median 84 days, interquartile range 77–91) (Voysey et al., 2021a), that means that booster doses occurred between the end of August and the beginning of September (Voysey et al., 2021b). Therefore, at the two data cut‐off dates (4 November and 7 December 2020), LD/SD cohort was, respectively, analysed about 2 (September to 4 November) and 3 months (5 November to 7 December) after the booster dose (considering that full vaccine protection was assessed to be achieved 22 days after vaccination). During the longitudinal study, the frequency of infected individuals in control group was 2.2% in the first time period (data cut‐off date 4 November) and 1.53% in the second time period (between the two data cut‐off dates). Because in United Kingdom the frequency of infected individuals increased during the month of November, it suggests that the second time period was shorter than the first. Nevertheless, the frequencies of spontaneous infection were somehow similar (and comparable) between the two groups. In addition, during the time between the two data cut‐off dates, a similar trend of the relative risk of infection was observable for the asymptomatic (transmissible) infection, for which the vaccination in the LD/SD cohort reached a relative risk of 0.64 ([95% CI 0.28–1.48], it was 0.41 [95% CI 0.17–0.99] on 4 November 2020), suggesting that the booster dose provided a protection for 2 months after which the immune protection seems to start to wane. Instead, no waning of vaccine efficacy was detected between 22 and 90 days after a single SD, for which the relative risk of symptomatic infection remained stable (median 0.24 [95% CI 0.14–0.41]) until 90 days (3 months) after vaccination, despite a 34% reduction (GMR 0.66 [95% CI 0.59–0.74]) of anti‐SARS‐CoV‐2 spike IgG responses after 90 days from the peak at Day 28 (Ramasamy et al., 2021; Voysey et al., 2021a). Intriguingly, the relative risk of symptomatic infection after a single SD within 90 days (0.24 [95% CI 0.14–0.41]) was similar to that in the LD/SD group when evaluated at the second cut‐off date (7 December, 0.19 [95% CI 0.10–0.38]) and lower than those in the SD/SD groups (see Figure 1). In this regard, it is expected that the prime‐boost regimen can induce both higher levels of neutralising antibodies and longer time protection (for several months) than a single dose. Indeed, 28 days after the second dose (about 1 month before the first data cut‐off date), the anti‐SARS‐CoV‐2 spike IgG responses in LD/SD group were extremely high (median 39,670 AU·ml^−1^ [IQR 21,068–66,338] 9–11 week interval and 49,584 AU·ml^−1^ [IQR 31,122–81,163] ≥12 week interval for participants aged 18–55 years) compared with those induced after 28 days by single (standard or low) doses (single LD, median 6439 AU·ml^−1^ [IQR 4338–10,640] for participants aged 18–55 years) (Ramasamy et al., 2021; Voysey et al., 2021a), suggesting that the decrease of anti‐SARS‐CoV‐2 immune protection may not be due to the decline of anti‐SARS‐CoV‐2 antibodies. Because the protection induced by a single dose lasted for at least 3 months (despite a significant reduction of anti‐SARS‐CoV‐2 spike IgG responses) and at 28 days after the second dose (1 month earlier than the first data cut‐off, 4 November 2020), participants in the LD/SD cohort displayed an expression of anti‐SARS‐CoV‐2 spike IgG about fivefold higher than after a single (SD or LD) dose (Ramasamy et al., 2021; Voysey et al., 2021a). It is unlikely that the decreased vaccine efficacy at the second data cut‐off date (7 December 2020) depends on the concentrations of anti‐SARS‐CoV‐2 antibodies. Rather, the relatively low protective efficacy recorded during the month of November after the booster dose of ChAdOx1 nCoV‐19 vaccine (relative risk of symptomatic infection 0.33) compared with single SD analyses (relative risk of symptomatic infection 0.24) assessed between 22 and 90 days (for which most data were collected during the prime‐boost interval, i.e., probably between September and October) suggests that other factors might be at play late after the booster dose. Of note, most of vaccine efficacy data regarding single standard dose (S‐D) between 91 and 120 days (91‐120d) after administration were likely to have been collected at the cut‐off date (7 December) when several participants assessed between 22 and 90 days (S‐D 22‐90d) were excluded because they received the booster dose (Voysey et al., 2021a). Therefore, similarly to the LD/SD group at the second cut‐off date (7 December), the S‐D (91‐120d) group underwent the evolution of SARS‐CoV‐2 infection that occurred during the last period of analysis (between November and 7 December, see later) and vaccine efficacy in this group also wanes, reaching a relative risk of symptomatic infection of 0.68 (95% CI 0.19–2.42) (Voysey et al., 2021a), thus suggesting a common factor (possibly independent of anti‐SARS‐CoV‐2 antibody concentration) that led to a general reduction of ChAdOx1 nCoV‐19 vaccine efficacy in that specific temporal period. Figure 1 summarises the relative risk of symptomatic infection of different groups of vaccine participants with different dose interval analysed in two different time periods (September/October vs. November/7 December 2020).

It is known that the impairment of vaccine‐induced immune protection can be due to reduction not only in concentration but also in specificity of anti‐SARS‐CoV‐2 neutralising antibodies. Therefore, it is possible that loss of antibody recognition might be involved in the reduction of immune protection ‘in vivo’. In this regard, evaluation of anti‐SARS‐CoV‐2 spike IgG responses has been assessed using the ancestral spike protein, it is therefore possible that the emergence of SARS‐CoV‐2 variants with spike protein mutations may underlie the discrepancy between anti‐SARS‐CoV‐2 spike IgG responses and vaccine efficacy late during the vaccine trials. In this regard, the SARS‐CoV‐2 Alpha variant (also known as B.1.1.7 or UK variant), which carries several mutations including in the spike protein, started circulating in England in late September and became the dominant lineage in December (Leung et al., 2021). In the United Kingdom, the proportion of the Alpha variant has increased from 0.1% in early October to 49.7% in late November among sequences available at 19 December 2020 (Leung et al., 2021), suggesting a cause–effect relationship between expansion of the Alpha variant and a possible decrease of efficacy of ChAdOx1 nCoV‐19 vaccine against symptomatic infection, during the two data cut‐off dates of the reports (5 November 2020 and 7 December 2020).

The Alpha variant contains eight spike protein mutations, in addition to D614G, including one mutation (N501Y) in the receptor binding domain (RBD), two deletions (69‐70del and 144del) in the N‐terminal domain (NTD) of the spike and one mutation (P681H) near the furin cleavage site (Davies, Abbott et al., 2021; Shen et al., 2021; Wang et al., 2021). However, the Alpha variant seems susceptible to neutralising antibodies elicited by ancestral spike vaccines (Shen et al., 2021), rather it has an enhanced binding to ACE2, a higher reproduction and an increased transmission that gives it a competitive advantage in humans (Davies, Abbott et al., 2021; Shen et al., 2021). Nevertheless, neutralisation by serum samples from recipients of vaccines with ancestral spike was moderately reduced, and a subset of monoclonal antibodies to the RBD of the spike protein is less effective against the Alpha variant (Shen et al., 2021), raising the possibility of a moderately increased risk of infection and virus transmission after vaccination with ancestral spike sequences. Consistent with this possibility, another report found that the Alpha variant was refractory to neutralisation by most monoclonal antibodies to the NTD of the spike protein and relatively resistant to a few monoclonal antibodies to ancestral RBD, which could cause escape from neutralising antibody control in vivo, thus threatening the protective efficacy of current vaccines (Wang et al., 2021). In this regard, a recent sequencing of the Alpha variant (Wise, 2021) revealed the presence of the E484K mutation (first identified in South Africa) and several studies showed reduced neutralising activity of monoclonal antibodies from convalescent or vaccinated individuals against virus mutants containing the E484K mutation (Chen et al., 2021; Madhi et al., 2021; Wibmer et al., 2021; Zhou et al., 2021). Moreover, the presence of the N439K mutation, which has emerged independently in several variant lineages, has been shown to increase both spike binding affinity for human ACE2 and resistance to several anti‐SARS‐CoV‐2 neutralising antibodies, which give SARS‐CoV‐2 variants carrying N439K a selective advantage (Thomson et al., 2021). Altogether these observations suggest that neutralising antibodies elicited by ancestral spike vaccines induce cross‐protection from the Alpha variant, although they may not be able to fully protect against the UK variant and, in particular, against its transmission. In this regard, a post hoc analysis of the efficacy of ChAdOx1 nCoV‐19 vaccine against the Alpha variant has shown that clinical efficacy against symptomatic infection was 70.4% (95% CI 43.6–84.5) (whereas it was 81.5% [95% CI 67.9–89.4] for non‐Alpha lineages) and it was 28.9% (95% CI −77.1 to 71.4) against asymptomatic infection (Emary et al., 2021); values that are very similar to those calculated in the present report for the LD/SD group between 5 November and 7 December, which were, respectively, 67.2% (95% CI 23.0–86.0) and 35.9% (95% CI −47.6 to 72.2). In line with these observations, the report showed that the neutralisation activity of vaccine‐induced antibodies in a live‐virus neutralisation assay against the Alpha variant was about nine times lower than against the ancestral lineage (GMR 8.9 [95% CI 7.2–11.0]) (Emary et al., 2021). Notably, participants of the study were recruited between 31 May and 13 November 2020 (Emary et al., 2021) which was before the Alpha variant expanded and evolved acquiring new immune escape mutations, thus suggesting that current vaccine protection could be even lower, in particular that of the single dose (see Figure 1). Altogether, these observations suggest that ChAdOx1 nCoV‐19 vaccine may not be able to block the transmission of the UK variant as efficiently as it blocks the transmission of the ancestral virus. Whether this possibility may occur only with ChAdOx1 nCoV‐19 vaccine or also with other vaccines based on the ancestral spike sequences is still unclear, although probable.

## 4 THE POSSIBLE EFFECTS OF EMERGING SARS‐CoV‐2 VARIANTS ON VACCINE EFFICACY, SARS‐CoV‐2 INFECTION AGE‐DISTRIBUTION AND SEVERITY, AND THE NEED TO STILL MAINTAIN PHYSICAL PREVENTIVE MEASURES

Because no hospital admissions or severe cases were reported in the ChAdOx1 nCoV‐19 arm (Voysey et al., 2021a, 2021b), the data clearly show that ChAdOx1 nCov‐19 is still effective against severe and persistent disease, during the emergence of the UK variant. It is clear that the number of both infected individuals and days of infectivity (related to severity) per person substantially influences the probability of both virus transmission and mutation (i.e. generation of variants). Therefore, at the moment, ancestral spike‐based vaccines are able to reduce the severity of symptoms and the time of infectivity and transmissibility of the UK variant. However, care should be taken because asymptomatic infection in vaccinated individuals may still spread the variant, albeit at lower efficiency. Indeed, if the vaccinated individuals do not maintain everyday preventive measures (such as the physical distancing and the use of face masks), they might turn into potential spreaders not only to uninfected and unvaccinated individuals, but potentially also to some asymptomatic individuals (during the first wave), which are instead more susceptible to new and highly infectious SARS‐CoV‐2 variants (e.g., Alpha/B.1.1.7). For example, SARS‐CoV‐2 spike variants with increased binding affinity to human ACE2 (such as N439K variants) also increases the probability of infecting a higher number not only of cells in a patient but also of individuals in a population. Therefore, they can produce a worse and persistent infection in a broader range of humans that finally provides an increased probability not only of transmission but also of generation of variants. Indeed, under the selective pressure of the immune system in convalescent and/or vaccinated people, adaptation processes of mutable RNA viruses (such as SARS‐CoV‐2 and influenza) constantly generates a heterogeneous pool of SARS‐CoV‐2 variants, which are continuously tested and selected in vivo in order not only to escape immune responses, antibody treatments and herd immunity but also to overcome ‘adverse’ environmental conditions and physical barriers (preventive measures). Fortunately, the nature of the new vaccine technology will rapidly allow for new vaccine variants with specific mutations. However, it is not clear how many vaccinations with different vaccine variants will be necessary before the pandemic is halted (and the global economy is recovered) and what will be the short‐ and long‐term consequences in efficacy and side effects of repeated vaccination.

In this context, yearly viral challenge of influenza virus is a good model to try to predict the effect of repeated exposures to mutant viruses and seasonal vaccine variants. Although influenza vaccines have successfully controlled the severe forms of infection, some previous infections and/or vaccinations with influenza strains can be sometimes counterprotective (Francis et al., 2019). Interactions between the immune system and mutant pathogens and/or vaccine variants are dynamic processes, which evolve at each exposure on the basis of previous host–pathogen interactions ‘memorised’ by the immune system of each individual and, by extension, of each population or community (Francis et al., 2019). The imprinting event of first influenza infection or of first vaccination generates a pool of long‐lasting immunological memory cells which remains throughout life and determines the response to subsequent infections or vaccinations in terms of both protection and adverse effects. It has been suggested that an elevated antigenic diversity between previous and subsequent vaccination permits the generation of new immune memory cells that provide more efficient protection against new viral variants. Conversely, repetition of antigenically related vaccines and previously existing low avidity antibodies derived from memory cells can lead to a deleterious outcome of a subsequent infection by causing disease enhancement (Francis et al., 2019). Therefore, the cumulative effects of repeated influenza virus infections and/or vaccinations can ‘unpredictably’ shape future immune responses that could be either beneficial or deleterious (Francis et al., 2019). In terms of repeated SARS‐CoV‐2 spike vaccinations, there is a further aspect of unpredictability due to the fact that influenza vaccines include inactivated influenza vaccine, live attenuated influenza vaccine or recombinant protein influenza vaccine. In contrast, the SARS‐CoV‐2 vaccines that have recently received emergency use authorisation in Europe include lipid nanoparticle‐encapsulated mRNA‐based vaccines or adenovirus‐vectored DNA‐based vaccines. When compared with traditional vaccines that use dead or weakened forms of the viruses, these new vaccines have an important difference in the envelope that contains the genetic material. The envelope is the vector that determines not only anti‐envelope/anti‐vector immune responses but also the cells in which the genetic content is inserted and expressed, the vaccine tropism. Differently from traditional vaccine platforms, these novel vaccine strategies induce anti‐envelope/anti‐vector immune responses that do not generate antiviral memory cells and insert the spike nucleotide sequence into cells independently of ACE2 expression, possibly driving a non‐specific immune response against cells that will never be infected by SARS‐CoV‐2 and/or possibly inserting new nucleic acid sequences into the delicate reproductive systems with potential consequences for the future generations (see Gonzalez et al., 2021). Therefore, in order to reduce current pressure on healthcare systems, vaccination should be focused on protecting the most vulnerable (minority) part of the population for which the risk/benefit balance of vaccination is more favourable.

At the same time, this vaccination strategy (successfully applied for the highly mutable influenza RNA virus, for which we have never attempted or needed to reach a herd immunity) is likely to limit vaccine‐driven immune selection pressure that, under current conditions of very high levels of virus replication and diffusion, may push to select SARS‐CoV‐2 ‘escaping’ variants. Most notably, the accidental ability of virus variants not only to produce persistent infections in a broader number of individuals including young and healthy people (who are relatively resistant to ancestral infection) but also to ‘survive’ in different environmental conditions (e.g. in different seasons and/or under physical preventive measures) provides a higher probability of transmission and a competitive advantage. In this regard, SARS‐CoV‐2 variants, which are more resistant to summer temperatures, humidity and UV rays, are already present in South Africa, Brazil, Chile and India, countries in which the variants emerged during their summer/wet seasons. Moreover, of particular concern are variants that are able to generate a persistent immune system response against viral infection in people with strong immune responses (such as young healthy people). In this regard, during the second wave of SARS‐CoV‐2 (September 2020 to 7 January 2021), there were more people (and in a shorter time period) in England's hospitals with COVID‐19 (weekly incidence per 100,000 inhabitants was 19.3 cases, calculated using the 2019 population estimates for the England, available from the UK National Statistics) than in the first wave (March to September 2020, weekly incidence per 100,000 inhabitants was 6.4 cases), indicating the higher infectivity of the UK SARS‐CoV‐2 variant (see Roxby, 2021). In particular, there was a relative increase in hospitalisation rates in younger age groups (1.72‐fold increase for the <17‐year age group) compared with the older age groups (1.35‐fold increase for the >65‐year age group), whereas relative increase was intermediate (1.46‐fold) for the 18–64‐year group (see Roxby, 2021). During the first wave of SARS‐CoV‐2, the prevalence of hospitalisation for COVID‐19 was one young patient (in the <17‐year age group of 12,023,568 individuals based on the 2019 population estimates for the England) for every 64 elderly patients (in the >65‐year age group of 10,353,716 individuals based on the 2019 population estimates for the England), that is, a relative risk ratio 0.016 (99% CI 0.015–0.017), whereas it significantly increased to one for every 50 elderly patients, that is, relative risk ratio 0.020 (99% CI 0.019–0.021) in the second wave, thus leading to a substantial decrease of the median age of hospitalised patients compared with the first wave. In line with this observation, a recent report observed a shift in the age composition, with significantly more UK variant cases among individuals aged 0–19 and significantly fewer UK variant cases among individuals aged 60–79, compared with non‐UK variant cases (Volz et al., 2021).

At this time, it is not possible to predict whether (spontaneous and vaccine‐driven) immune pressure could quickly induce a mild endemic disease or whether this could occur over the course of years, passing through a more aggressive and severe disease. In this regard, a recent report estimated that infection with a new variant of the Alpha lineage spread in the United Kingdom during December 2020 has the potential to cause substantial additional mortality, compared with previously circulating variants (54,906 matched pairs of participants between 1 October 2020 and 29 January 2021), increasing the probability of risk of mortality from 2.5 to 4.1 per 1000 detected cases (Challen et al., 2021). Results are in agreement with those of another recent report (Davies, Jarvis, et al., 2021) and suggest that, at the moment, the progression of the disease is becoming worse. Moreover, the accidental ability of reinfection or of infection of vaccinated individuals provides a competitive advantage to some SARS‐CoV‐2 variants, particularly in highly vaccinated countries, in which most people are fully resistant to the ancestral virus. If the vaccinated population become susceptible to a variant infection, this variant will have plenty of subjects to infect again, potentially leading to a ‘rebound’ effect in highly vaccinated countries (as it may occur, e.g., in Chile and/or in the United Kingdom and/or in Israel; see Chambers, 2021; Schraer, 2021). Such an eventuality, in our globalised world, will potentially spread the new variant to less vaccinated (less privileged) countries (potentially turning ‘vaccinated’ countries and individuals into potential spreaders that might lead to a sort of an involuntary biological world war). In this regard, young vaccinated individuals, which can develop asymptomatic infection, owning the so‐called ‘GreenPass’ might become important carriers that may spread infection again, finally generating more drug/vaccine‐resistant variants, as it occurs with the abuse of antibiotics.

After consideration of all these possibilities, it is clear that, although vaccine strategies may temporarily reduce both disease severity and spread, they are unlikely to prevent the appearance of new variants and to be effective in quickly solving the pandemic crisis. It is instead likely that mild endemic disease will be slowly achievable by mass vaccination and neutralising antibody strategies, whereas global herd immunity (as for the influenza virus) is unlikely to be attained. On the other hand, it is not clear how mass vaccination (never undertaken against highly mutable RNA viruses) may influence the course of the SARS‐CoV‐2 mutant evolution, although this information will rapidly be available in highly vaccinated countries. Therefore, more caution should be taken with vaccination strategies in order to avoid both the development of more aggressive mutants and health risks in young people, potentially inducible by repeated vaccination using the new type of vaccines. Again, vaccination strategy for influenza virus, which is limited to the most vulnerable people, has been shown to successfully control the severe infection and to not induce more aggressive variants. Finally, there is the need to keep searching for new pharmacological therapies, and more scientific efforts should be directed towards pharmacological approaches that, working downstream in the infection pathways, are independent of the virus variant and allow the development of a natural and lasting immunity. In this regard, clinical trials employing new safe pharmacological treatments for COVID‐19 with a potentially effective mechanism of action that are not tested in clinical trials yet, such as inhibitors of ACE2/ADAM17 zinc‐metalloprotease activity, are urgently needed (see Zamai, 2020, 2021; Yuan et al., 2020).

### 4.1 Nomenclature of Targets and Ligands

Key protein targets and ligands in this article are hyperlinked to corresponding entries in the IUPHAR/BPS Guide to PHARMACOLOGY (http://www.guidetopharmacology.org) and are permanently archived in the Concise Guide to PHARMACOLOGY 2019/20 (Alexander, Fabbro et al., 2019; Alexander, Kelly et al., 2019).

## AUTHOR CONTRIBUTIONS

L.Z. conceived of the article and wrote it. M.B.L.R. elaborated the statistical analyses and corrected the manuscript. L.Z. and M.B.L.R. read and approved the final manuscript.

## CONFLICT OF INTEREST

The authors declare no competing interests.

## Data Availability

Data sharing is not applicable to this article because no new data were created or analysed in this study.
